# Single-cell resolution spatial transcriptomic signature of the retrosplenial cortex during memory consolidation

**DOI:** 10.1038/s41380-025-03331-3

**Published:** 2025-11-04

**Authors:** Savannah R. Bliese, Budhaditya Basu, Stacy E. Beyer, Muhammad Elsadany, Jacob J. Michaelson, Snehajyoti Chatterjee

**Affiliations:** 1 Department of Neuroscience and Pharmacology, Carver College of Medicine, University of Iowa, Iowa City, IA 52242 USA; 2 Iowa Neuroscience Institute, University of Iowa, Iowa City, IA 52242 USA; 3 Interdisciplinary Graduate Program in Neuroscience, University of Iowa, Iowa City, IA 52242 USA; 4 Department of Psychiatry, University of Iowa Hospitals and Clinics, Iowa City, IA 52242 USA

**Keywords:** Neuroscience, Molecular biology

## Abstract

The retrosplenial cortex (RSC) is a critical brain region that is activated during spatial memory tasks and plays a crucial role in the consolidation of long-term memory. Various classes of RSC excitatory neurons across different laminar layers serve as the central hub for neuronal connections between the RSC and other brain regions, such as the hippocampus. Despite the established role of the RSC in spatial memory, the transcriptomic signature of the neuronal subtypes in the RSC during spatial memory consolidation remained elusive. Here, we used unbiased and targeted spatial transcriptomics to identify the RSC transcriptional signature after a spatial memory task. Genes related to transcription regulation, protein folding, and mitogen-activated protein kinase pathways were upregulated in the RSC during an early time window of memory consolidation. Furthermore, cell-type and excitatory neuronal layer-specific changes in gene expression were resolved using Xenium spatial transcriptomics. A deep learning computational tool uncovered cell-type-specific molecular activation patterns within the RSC after learning. Conversely, in a mouse model of Alzheimer’s disease and related dementia (ADRD) exhibiting tau hyperphosphorylation in the RSC, there was a reduction in predicted neuronal activation following learning. Notably, learning-induced *Fos* expression was decreased in excitatory neurons of the RSC in the ADRD mice. Finally, we observed that blocking RSC excitatory neurons during the early temporal window after learning using a chemogenetic approach impaired long-term spatial memory in adult mice. Our results reveal a molecular signature of the RSC after learning and emphasize the role of RSC excitatory neurons during spatial memory consolidation.

## Introduction

The retrosplenial cortex (RSC) is a neocortical structure that acts as an integration center between sensory, limbic, and higher-order cortical brain regions [1]. In humans, the RSC has been shown to be integral for topographical and episodic memories [1, 2]. The RSC has also been identified as a key structure for spatial [3–8] and contextual fear memories [9–11] in rodents. The RSC integrates spatial navigational information using place-like and head-direction cells [12], and damage to the RSC leads to deficits in spatial cognition [2]. Furthermore, the RSC has been linked to numerous neurological disorders, including post-traumatic stress disorder [13], schizophrenia [14, 15], and Alzheimer’s Disease and related dementias (ADRD) [16, 17]. RSC dysfunction is observed during the early stages of ADRD, and lesions in RSC lead to memory impairments [18–22]. Additionally, neuronal tracing studies have identified several major RSC connections to other brain regions, including the hippocampus and entorhinal cortex [23–25]. These regions are critical within the spatial memory circuit, and the connectivity between the RSC and these regions indicates its essential role in spatial navigation and memory [26]. Spatial gene expression changes in the dorsal hippocampus during an early time window after learning are essential for long-term memory consolidation [27, 28]. However, the spatial transcriptional signature of the RSC during this early time point of memory consolidation has not been clearly described.

Recent single-cell transcriptomic studies have identified multiple spatially restricted excitatory neuronal sub-types within the RSC, many of which are unique to this region in comparison to other cortical areas [29, 30]. The RSC is anatomically diverse, comprising of anterior and posterior as well as granular and agranular subregions. These subregions receive distinct inputs, project to a wide variety of brain regions [31, 32], and display differing roles in behavior. Glutamatergic neurotransmission in the RSC has been implicated in contextual memory consolidation [9, 10, 33–35]. Furthermore, these excitatory neurons within the RSC laminar layers exhibit functional and behavioral differences; for example, ablation of neurons in layers 4 and 5 of granular RSC caused amnesia in rats [36]. But, given this diversity, it has been challenging to delineate specific roles for each excitatory neuronal subtype within the RSC laminae. Therefore, it is critical to investigate the molecular signature of layer-specific patterns of excitatory neurons in the RSC to determine which neuronal populations are critical for long-term memory.

The molecular mechanism underlying spatial memory consolidation involves precisely timed transcriptional events. Memory-encoding neuronal ensembles comprising cells exhibiting the induced expression of immediate early genes (IEGs), also known as engram cells, have been extensively studied in the forebrain [37, 38]. Studies have shown the upregulation of activity-induced genes such as *Arc* and *Fos* in the RSC after learning in a contextual fear conditioning task [39, 40]. Using in vivo IEG imaging, a recent study showed engram activation in the RSC during a spatial memory task [41]. The study also demonstrated that the stability of the RSC engrams recruited during a spatial navigation task is associated with memory performance, providing evidence for a relationship between spatial memory and RSC engram ensembles [41]. Bulk transcriptomic profiling of the RSC and hippocampus identified a common induction pattern of an engram-specific gene expression signature after spatial learning in adult mice [42]. A study on the expression of Fos, an engram marker, suggests a unique role the RSC plays in tracking an animal’s position in the environment [43]. However, such learning-induced gene expression in the RSC at single-cell resolution across excitatory neuronal layers has never been performed. Therefore, understanding the precise transcriptomic signature of IEG that are markers of engrams in the RSC will provide mechanistic insights into the role of the RSC in spatial memory consolidation.

Single nuclei RNA sequencing and spatial transcriptomics have been used to study RSC-specific cell type identity [29] and gene expression following nerve injury [44]. However, the literature lacks single-cell and spatial transcriptomic studies investigating the anatomically distinct RSC neurons following a learning experience. In this study, we used cutting-edge molecular approaches to delineate the transcriptional signatures within the RSC during spatial memory consolidation. First, we  investigated the unbiased transcriptional signature in the RSC from our previously published spatial transcriptomics dataset after spatial learning. Next, we utilized a newly developed spatial transcriptomics platform, Xenium, to investigate learning-induced gene expression in the RSC at single-cell resolution following spatial learning in adult mice. We utilized a computational tool to predict the neuronal activity pattern following learning in the RSC of adult mice. We next examined how tau pathology impacts the predicted neuronal activity after learning in a mouse model of ADRD. Lastly, using a chemogenetic approach, we demonstrate the importance of learning-induced activation of excitatory neurons in the RSC for long-term spatial memory consolidation. Together, our findings provide insights into the transcriptomic signature of the RSC at single-cell resolution during long-term memory consolidation.

## Results

### Learning in a spatial object recognition (SOR) task induces immediate early gene expression changes in the RSC

**Fig. 1:**
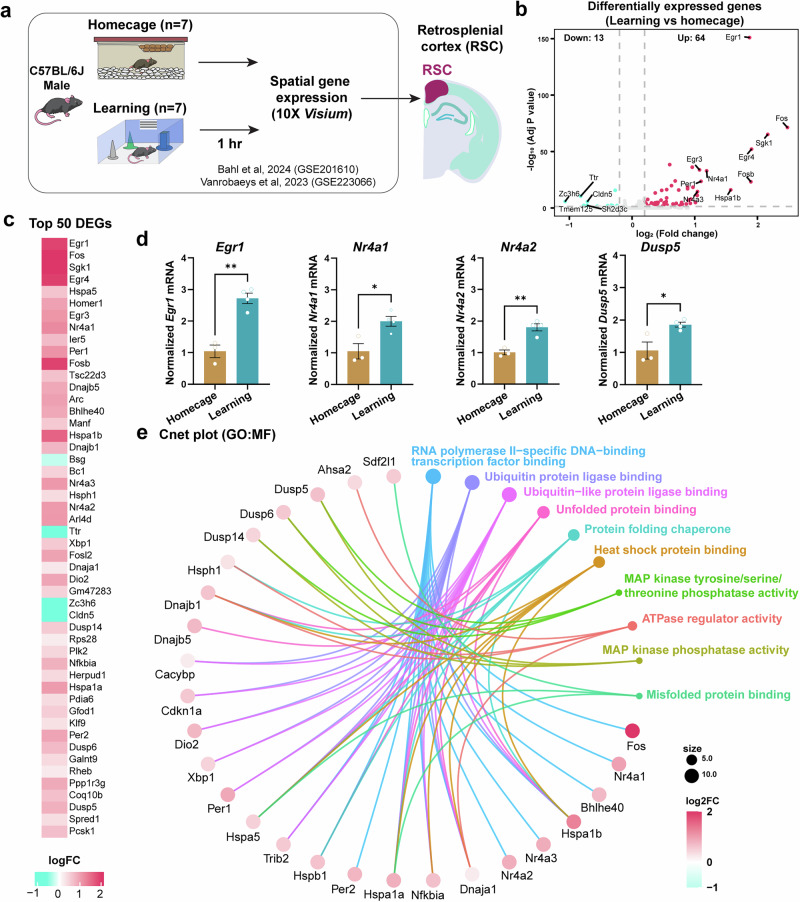
Spatial transcriptomic analysis using Visium reveals learning-induced gene expression in the RSC. **a** Schematics of the experimental paradigm being utilized for spatial gene expression and the region of interest being analyzed. **b** Number of differentially expressed genes found within the RSC 1 h after learning (n = 7 for both groups). **c** Heatmap displaying the top 50 significant differentially expressed genes within the RSC. **d** Expression of *Egr1*, *Nr4a1*, and *Dusp5* within the RSC 1 h after learning. Normalized to homecage animals (homecage n = 3, and learning n = 4). Unpaired t-test: *p < 0.05 (Nr4a1: p = 0.0178, Dusp5: 0.0198), **p < 0.005 (Egr1: p = 0.0013). The data is shown as group mean ± SEM. **e** Cnet plot exhibiting gene ontology (molecular function) enrichment analysis of the differentially expressed genes that were identified within the RSC.

RSC neuronal activation has been reported during tasks that utilize spatial locations and landmarks [45–48]. Interestingly, introducing an object to the environment alters the mean firing rate of RSC neurons [48]. More specifically, activity within the anterior RSC has been shown to be required for long-term spatial memory [49, 50]. Therefore, we first investigated the unbiased transcriptomic signature of the anterior RSC following learning in a spatial object recognition (SOR) task from our previously published datasets (GSE223066 and GSE201610) [27, 51]. Using these datasets, we have previously demonstrated the spatial transcriptomic signature of the dorsal hippocampal subregions one hour after training in the SOR task [27]; however, the learning-induced gene expression signature in the RSC was not studied. To investigate the gene expression changes in the RSC after spatial learning, we performed differential expression of genes (DEG) analysis on the RSC region comparing learning with homecage controls, and this analysis revealed 64 upregulated and 13 downregulated genes (Fig. 1a, b, and Supplemental Table 1). Several of the top significant genes identified were IEGs such as *Fos*, *Arc*, *Nr4a1*, *and Egr1* (Fig. 1c). These activity-induced genes have been well characterized for their roles in learning and memory in the hippocampus and are used as markers of neuronal activity and engram ensembles [27, 39]. We further validated the induction of a few IEGs after learning from an independent experiment using qPCR analysis from RSC tissue (Fig. 1d). Next, we performed a gene ontology (GO) enrichment analysis to identify the molecular functions most represented among the DEGs in the RSC following learning (Supplemental Table 2). The top enriched pathways in the RSC include RNA polymerase II-specific DNA binding transcription factor binding, ubiquitin-protein ligase biding and ubiquitin-like protein ligase binding, unfolded protein binding, protein chaperone, heat shock protein binding, MAP kinase activity, MAP kinase phosphatase activity, ATPase regulator activity and misfolded protein binding (Fig. 1e). Genes related to DNA binding transcription factor binding such as *Nr4a1*, *Nr4a2*, and *Nr4a3* are closely linked to long-term memory consolidation and mice expressing a dominant negative form of Nr4a that blocks the transactivation function of all the Nr4a family members in the excitatory neurons in forebrain exhibits long-term memory deficits in contextual fear conditioning [52] and SOR tasks [53].

### Single-cell resolution spatial transcriptomic analysis reveals distinct and overlapping learning-induced gene expression changes across major cell types in the RSC

**Fig. 2:**
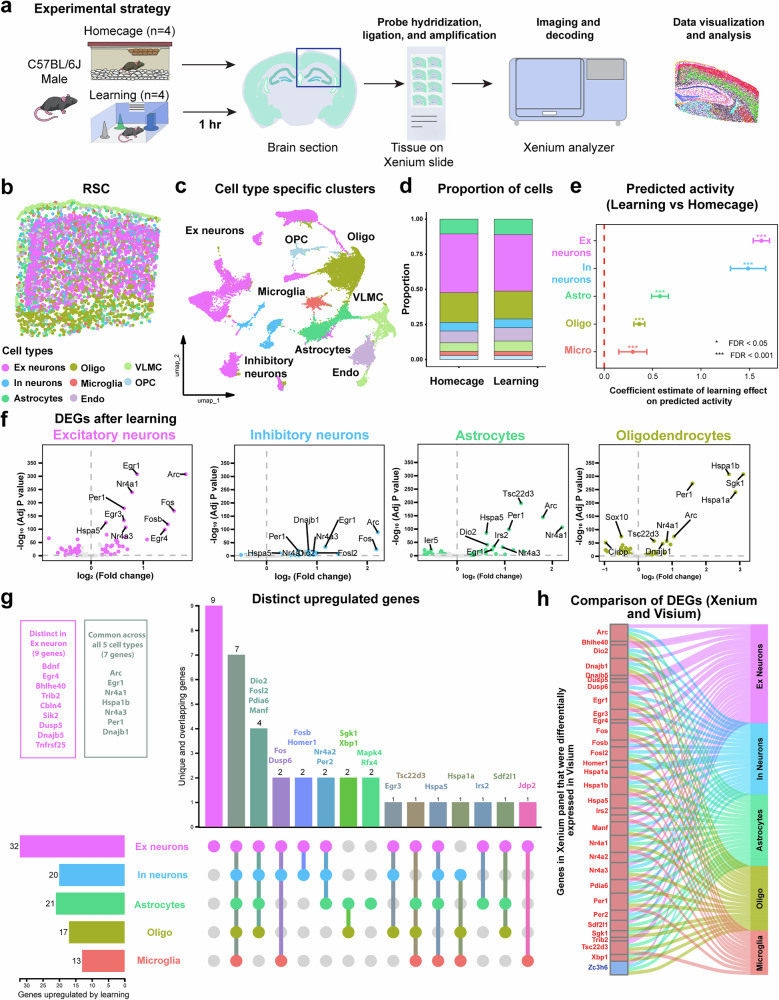
Single-cell resolution spatial transcriptomic approach reveals learning-induced gene expression across major cell types in RSC. **a** Schematics depicting the spatial learning task, the region of interest collected, and the processing for Xenium pipeline. **b** Spatial position of cells of a single coronal tissue section colored by major cell types in RSC. Ex neurons = Excitatory neurons, In neurons = Inhibitory neurons, Oligo = Oligodendrocytes, Endo = Endothelial cells, VLMC = Vascular leptomeningeal cells, OPC = Oligodendrocyte progenitor cells **c** UMAP plot displaying unique cell types identified within the RSC. **d** Bar plot comparing the proportion of cell types found among the two groups (homecage, n = 4 and learning, n = 4). **e** Logistic regression results for predicting experimental groups using NEUROeSTIMator predicted activity. The colored dots represent the ß coefficients for predicted activity in the learning vs homecage experiment across major cell types. **f** Volcano plots demonstrating the top significant differentially expressed genes for the excitatory, inhibitory neuronal cell types, astrocytes, and oligodendrocytes. The genes with FDR < 0.05, absolute log_2_ fold change > 0.2 were considered significant and colored by cell type identity. **g** Upset plot illustrating the unique and overlapping upregulated genes across the major cell types identified within the RSC. **h** Sankey plot shows the common genes in both Xenium and Visium experiment that were significantly differentially expressed (red: upregulated, blue: downregulated) in the major cell types identified in Xenium analysis.

Our unbiased gene expression analysis using the spatial transcriptomics (Visium) approach revealed a learning-induced gene expression signature in the RSC (Fig. 1). However, this approach lacks information regarding which cell types exhibit these learning-induced gene expression changes. Therefore, to determine the precise cell type-specific gene expression signature of the RSC, we utilized Xenium, an in situ- hybridization approach for single-cell analysis of each RNA molecule at a high spatial resolution within anatomically distinct tissue regions. We trained adult male mice in the SOR task and collected brains one hour after training for Xenium analysis. Homecage animals were used as baseline controls (Fig. 2a). We employed a 297-gene panel composed of a standard probe panel including cell-type markers, genes responsive to learning and memory, and genes involved in other processes (full probe list: Supplemental Table 3). We obtained 26,484 high-quality cells from coronal sections in the RSC across learning and homecage animals.

Within the RSC, we used the pre-defined marker gene expression in the Xenium gene panel to identify the different cell types. We identified eight major cell types within the RSC, including excitatory neurons, inhibitory neurons, astrocytes, oligodendrocytes, endothelial cells, vascular leptomeningeal cells (VLMCs), microglia, and oligodendrocyte progenitor cells (OPCs) (Fig. 2b, c and Supplementary Fig. 1). The proportion of cells in each of the clusters between homecage and learning samples was comparable between the two groups (Fig. 2d). Given the role of neurons (excitatory and inhibitory) and glial cells (astrocytes [54], oligodendrocytes [55], and microglia [56]) in memory consolidation, we primarily focused our analysis on these five major cell types in the RSC. We applied a deep learning computational model, NEUROeSTIMator [51], to identify activation patterns of RSC cells following learning in the SOR task. The NEUROeSTIMator analysis predicted higher activity in all cell types in the learning group compared to the homecage control group. Highest increases in predicted activity were observed in excitatory and inhibitory neurons following SOR learning (Fig. 2e). Next, we investigated the differentially expressed genes following learning in these five major RSC cell types. Among the 297 genes analyzed from the Xenium panel, a comparison between learning and homecage groups revealed 96 DEGs in excitatory neurons (32 up and 64 down), 23 DEGs in inhibitory neurons (20 up and 3 down), 43 DEGs in astrocytes (21 up and 22 down), 44 DEGs in oligodendrocytes (17 up and 27 down), and 14 DEGs in microglia (13 up and 1 down) (Fig. 2f, Supplementary Fig. 2, and Supplemental Table 4). Further, Gene Ontology (GO: molecular function) enrichment analysis revealed RNA pol II-specific DNA-binding transcription factors to be the most represented function across the five major cell types (Supplementary Fig. 3 and Supplementary Table 5). Using an UpSet plot, we examined the distinctly upregulated and downregulated genes after learning within these cell types in the RSC. Among the upregulated genes, *Arc*, *Egr1*, *Nr4a1*, *Hspa1b*, *Nr4a3*, *Per1*, and *Dnajb1* were induced after learning in all the five major cell types (excitatory and inhibitory neurons, astrocytes, oligodendrocytes, and microglia), while *Bdnf*, *Egr4*, *Bhlhe40*, *Trib2*, *Cbln4*, *Sik2*, *Dusp5*, *Dnajb5*, and *Tnfrsf25* were induced exclusively in excitatory neurons. We also found that neuronal activity response genes *Fosb* and *Homer1* were exclusively upregulated in neurons, while *Sgk1* and *Xbp1* were exclusively upregulated in oligodendrocytes and astrocytes (Fig. 2g). Among the genes downregulated after learning, *Cirbp*, *Dner*, *Dpy19l1*, *Lypd6*, *Slc17a7* and *Garnl3* were downregulated in excitatory neurons, astrocytes, and oligodendrocytes. *Id2*, *Igfbp5*, *Parm1*, *Cdh20*, *Nrep*, and *Hpcal1* were downregulated in excitatory neurons, and oligodendrocytes. *Rbm3* was downregulated in excitatory neurons, inhibitory neurons, and oligodendrocytes. *Zc3h6* was downregulated in excitatory and inhibitory neurons, astrocytes, and oligodendrocytes, while *Plekha2* was downregulated in oligodendrocytes and microglia (Supplementary Fig. 4). Zinc finger CCCH containing 6 (Zc3h6) is predicted to have RNA binding and metal ion binding activity [57]. However, very little is known about Zc3h6 and its function in the brain. Comparing the learning-responsive DEGs from Visium and Xenium suggests that neurons in the RSC showed the highest overlap of learning-induced genes, particularly the upregulated genes in excitatory neurons (Fig. 2h).

To further identify the gene expression signature of active neurons after learning, we separated RSC excitatory and inhibitory neurons into active and non-active states based on *Fos* expression levels in the learning group. DEG analysis comparing Fos^+^ and Fos^-^ excitatory neurons in the RSC revealed 42 DEGs, and only 7 DEGs in inhibitory neurons in the RSC (Supplementary Fig. 5, Supplementary Table 6). We found that the classical immediate-early genes *Fos*, *Fosb*, *Nr4a1*, *Nr4a2*, *Bdnf*, *Egr1*, *Egr4*, and *Dusp6* were upregulated in Fos^+^ excitatory neurons compared to Fos^-^ neurons in the learning group. *Fos*, *Fosb*, *Fosl2*, and *Egr1* were upregulated in Fos^+^ inhibitory neurons (Supplementary Fig. 5). Thus, our spatial transcriptomic analysis using Xenium revealed a cell-type-specific signature of learning-induced genes in the RSC.

### Learning induces IEG expression changes across layer-specific neurons of the RSC

**Fig. 3:**
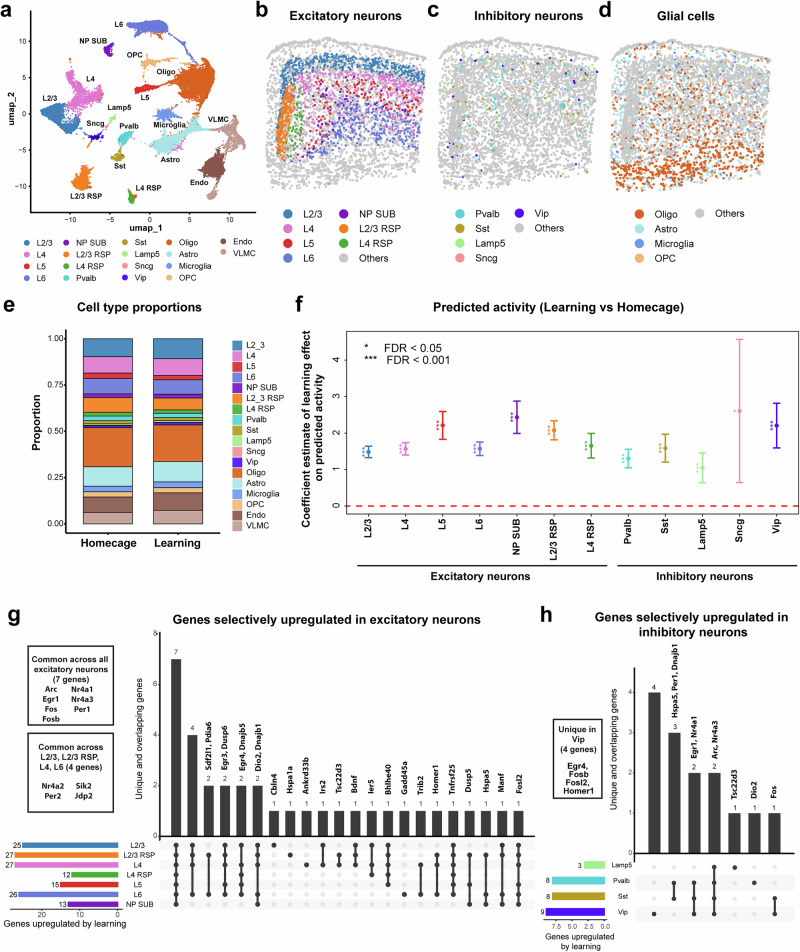
Single-cell resolution spatial transcriptomic analysis reveals the induction of IEGs after learning within different classes of RSC neurons. **a** UMAP exhibiting the layer-specific neuronal subtypes identified within the RSC. **b–d** Spatial map of b) excitatory neuronal sub-types c) inhibitory neuronal sub-types and d) glial sub-types within RSC. **e** Bar plot comparing the proportion of all the cell types found among the two groups (homecage and learning) in RSC. **f** Logistic regression coefficients for NEUROeSTIMator-predicted activity across excitatory and inhibitory neuronal sub-types, comparing the learning group to homecage. **g**, **h** Upset plot showing the unique and overlapping expression patterns of upregulated genes across different g) excitatory neuronal sub-type and h) inhibitory neuron sub-types within the RSC.

Our learning-induced gene expression analysis in the RSC using the Visium platform identified a higher number of upregulated genes in the RSC than downregulated genes. Importantly, we found a robust learning-induced upregulation of genes commonly used as engram markers in the RSC. RSC excitatory neuronal sub-types exhibit spatially recognizable laminar structural features [1, 29]. Additionally, these layer-specific glutamatergic neurons show unique electrophysiological characteristics [58, 59] and receive distinct inputs from the hippocampus [60, 61]. We performed unsupervised clustering from both the groups (homecage and learning) to identify the neuronal sub-types localized across different layers in the RSC. Investigating the expression of marker genes within each cluster of excitatory neurons further allowed us to classify the major layer-specific excitatory neuronal sub-types (Supplementary Fig. 6 and 7). We identified seven major excitatory neuronal sub-types: layer 2/3 (L2/3), retrosplenial specific layer 2/3 (L2/3 RSP), layer 4 (L4), retrosplenial specific layer 4 (L4 RSP), layer 5 (L5), layer 6 (L6), and near projecting-subiculum (NP SUB) (Fig. 3a, b). Similarly, we also identified five major inhibitory neuronal populations (Pvalb, Sst, Vip, Lam5, and Sncg) along with glial cells (astrocytes, oligodendrocytes, microglia and OPC) in the RSC (Fig. 3a, c and d). The proportion of cells in each cluster, comparing homecage and learning samples, was comparable between the two groups (Fig. 3e). Further, NEUROeSTIMator analysis revealed higher predicted activity in all the RSC neuronal subtypes comparing learning and homecage (Fig. 3f), suggesting a global increase in neuronal activity following learning.

To examine the learning-induced gene expression within the different populations of neurons, we performed DEG analysis on the 297 genes from the Xenium panel on all the neuronal sub-types (Supplemental Table 7). We used an Upset plot to investigate the learning-induced genes upregulated in each of the seven major excitatory neuronal populations identified in the RSC (Fig. 3g). Genes upregulated in only one sub-type of excitatory neurons include *Cbln4* upregulated in L2/3, *Hspa1a* in L2/3 RSP, *Ankrd33b* in L4, and *Gadd45a* in L6. Memory-related IEGs *Arc*, *Egr1*, *Fos*, *Fosb*, *Nr4a1*, *Nr4a3*, and *Per1* were commonly upregulated across all the seven major excitatory neuronal cell types, while TNF-receptor superfamily-related gene *Tnfrsf25* was upregulated in all the excitatory neuronal cell types except NP SUB. *Nr4a2*, *Per2*, *Sik2*, and *Jdp2* were upregulated in L2/3, L2/3 RSP, L4 and L6. Unfolded protein binding factors *Dio2* and *Dnajb1* were upregulated in L2/3, L2/3 RSP, L4, L6, and NP SUB, while chaperones *Sdf2l1* and *Pdia6* were upregulated in L2/3 RSP and L6. MAPK pathway-related genes *Egr3*, *Dusp6* were upregulated in L2/3, L2/3 RSP, L4, L5 and L6. Brain-derived neurotrophic factor and memory-related gene *Bdnf* was upregulated in L2/3, L2/3 RSP, and L4, and immediate early response gene *Ier5* was upregulated in L2/3, L4, and L4 RSP (Fig. 3g, Supplemental Table 7). Among the inhibitory neurons, *Arc* and *Nr4a3* were commonly upregulated across all four inhibitory neuronal populations, while *Egr1* and *Nr4a1* were upregulated in Pvalb, Sst, and Vip inhibitory neurons. *Fos* was upregulated in Sst and Vip inhibitory neurons, and *Hspa5*, *Per1*, and *Dnajb1* were upregulated in Pvalb and Sst neurons (Fig. 3h, Supplemental Table 7). Therefore, our spatial transcriptomic analysis after learning suggests an overall induction of IEG expression in the major neuronal populations, particularly across excitatory neuronal layers.

#### Neuronal activation after learning is reduced in the RSC of a mouse model of ADRD

**Fig. 4:**
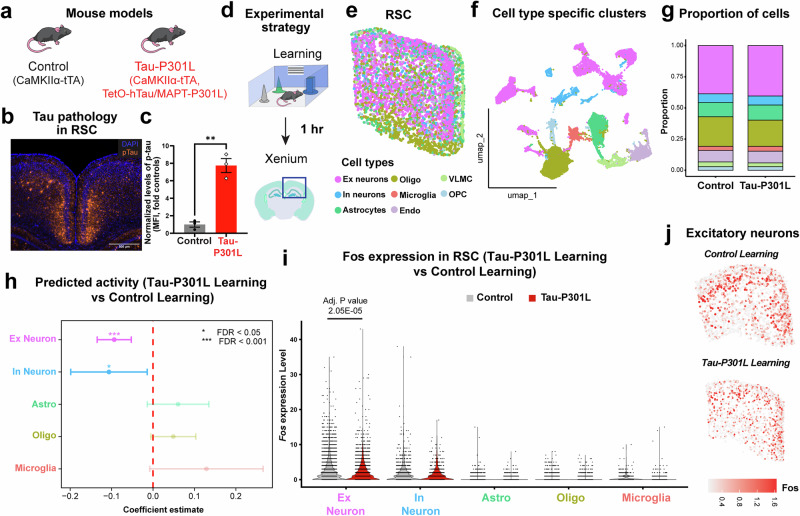
RSC of a mouse model of ADRD (Tau P301L) exhibit reduced neuronal activity following spatial learning. **a** Schematics depicting the control and Tau-P301L mice. **b** Immunofluorescence of the RSC region of Tau-P301L mice using an antibody that detects hyperphosphorylation of tau at Ser202, Thr205 (AH36). **c** Mean fluorescence intensity (MFI) of phosphorylated tau expression within the RSC. Unpaired T test: t (4) = 7.920, p = 0.0014. n = 3 for both control and Tau-P301L groups. The data is shown as group mean ± SEM. **d** Schematics depicting the spatial learning task, the region of interest collected, and the processing for Xenium pipeline. Both Tau-P301L (n = 3) and control (n = 4) mice were trained in the SOR task. **e** Spatial map of major cell types identified in the RSC. **f** UMAP plot shows clustering of cells that are colored according to the major cell type identity. **g** Bar plot comparing the proportion of major cell types in RSC found in both the control-learning and Tau-P301L-learning groups. **h** Logistic regression coefficient estimate for NEUROeSTIMator-predicted activity in Tau-P301L learning mice compared to control learning. **i** Violin plot showing expression of *Fos* across all major cell types between Tau-P301L and control mice after learning. Excitatory neurons show reduced *Fos* expression (Seurat Wilcox test, adjusted P value 2.05E-05, log2FoldChange: −0.23) comparing P301L and control mice. **j** Spatial map of *Fos mRNA* expression in the excitatory neurons of both control-learning and Tau P301L-learning groups.

Long-term memory impairment and hyperphosphorylation of microtubule-associated protein tau (MAPT) are common in patients with ADRD [62]. We have previously shown that a tauopathy model of ADRD (rTg4510 mice: Tau-P301L) that expresses mutant human tau in excitatory neurons shows spatial memory impairment in the SOR task [53]. An immunofluorescence analysis showed hyperphosphorylation of tau in the RSC of Tau-P301L (rTg4510) mice (Fig. 4a–c). Next, we performed spatial transcriptomic analysis using Xenium from the RSC of Tau-P301L and control mice after learning in the SOR task (Fig. 4d). Using the same gene panel as we used for the wild-type mice (Figs. 2, 3), we identified the major cell types in the RSC of Tau-P301L and control mice based on marker gene expression (Fig. 4e, f, Supplementary Figs. 8, 9). The proportion of different cell types was similar between the two experimental groups (Tau-P301L and control mice) (Fig. 4g). We then utilized the NEUROeSTIMator tool to predict activity in different RSC cell types. We found that excitatory and inhibitory neurons of Tau-P301L mice exhibited reduced activity after learning compared to controls (Fig. 4h). Additionally, *Fos* expression was downregulated only in excitatory neurons of Tau-P301L mice compared to controls (Fig. 4i, j). Taken together, our results suggest reduced activation of RSC excitatory neurons after learning in this tauopathy model of ADRD.

### Chemogenetic inhibition of the RSC excitatory neurons after learning impairs long-term spatial memory

**Fig. 5:**
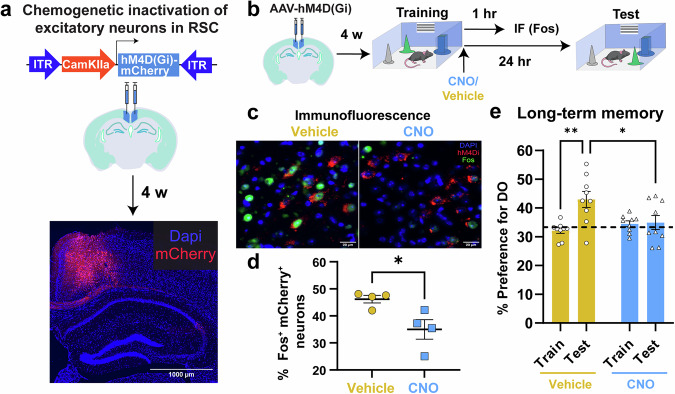
Activation of excitatory neurons after learning is essential for long-term spatial memory consolidation. **a** Graphic demonstration of the viral injection site, the viral construct that was used, and a representative immunofluorescence image showing the resulting viral expression within the RSC 4 weeks after infusion. **b** Schematic for the behavioral paradigm utilized. **c** Representative immunofluorescence images demonstrating c-Fos protein expression within DREADD positive neurons for the saline and CNO treated groups. **d** Dot plot showing the percent of DREADD positive neurons which are also positive for c-Fos protein expression 1 h after learning in an SOR task. Unpaired t test: t (6) = 2.894, p = 0.0276. n = 4 for both vehicle and CNO conditions. The data is shown as group mean ± SEM. Each yellow dot represents one animal in vehicle exposed group; each blue square represents one animal in CNO treated group. **e** Bar dot plot displaying behavioral performance on the SOR task between the saline and CNO injected animals. Long-term memory was assessed by finding the percent preference for the disturbed object (DO). 2 Way Anova: Significant trial type (Train-Test) x drug (vehicle-CNO) interaction: F (1, 16) = 7.205, p = 0.0163, main effect of trial type: F (1, 16) = 8.588, p = 0.0098. Šídák’s multiple comparisons test: vehicle: train vs test: p = 0.0022, test: vehicle vs CNO: p = 0.0145. n = 9 for both vehicle and CNO conditions. The data is shown as mean ± SEM. Each circle represents one animal in vehicle exposed group and each triangle represent one animal in CNO treated group.

Our spatial transcriptomics data identified robust learning-induced gene expression changes in the excitatory neurons of the RSC in wild-type mice. Further, the tauopathy model of ADRD showed reduced transcriptional signature of neuronal activation within the excitatory neurons after learning in RSC. To corroborate these findings, we used a chemogenetic approach to selectively manipulate the excitatory neurons immediately after learning, a critical time window for memory consolidation. We manipulated the activity of the excitatory neuronal population using a viral-based inhibitory designer receptor exclusively activated by designer drugs (DREADD) construct, which reduces the excitability of the neurons [63]. The expression of the construct was restricted within the RSC, but the CA2 region also displayed mCherry labeling, possibly due to viral affinity [64] or potential connectivity between RSC and CA2 (Fig. 5a). Following training in the SOR task, animals were injected with clozapine-n-oxide (CNO, 2 mg/kg) (Fig. 5b). The virally expressed receptors respond specifically to CNO, allowing us to selectively block learning-induced activation of these neurons during the early time point immediately after learning. CNO treatment reduced the percentage of neurons expressing Fos, a marker of engram ensembles, within mCherry positive neurons (excitatory neurons infected by the DREADD virus) one hour after training in SOR, suggesting reduced neuronal activation in the CNO treated group (Fig. 5c, d). Importantly, in homecage animals no differences were observed in the number of Fos positive neurons between saline and CNO conditions, indicating that the CNO treatment did not affect the activity level of the excitatory neurons at baseline (Supplementary Fig. 10). Given the importance of engram ensembles in memory allocation during this period after learning [65], we aimed to test the long-term spatial memory when excitatory neurons within the RSC are selectively manipulated during this early time window. We found that mice injected with CNO immediately after training showed no preference towards the displaced object in SOR during the 24-h long-term memory test (Fig. 5e). In contrast, mice injected with vehicle (saline) showed increased preference towards the displaced object during the test session compared to CNO injected mice (Fig. 5e). This finding suggests that reduced RSC excitatory neuronal activity during memory consolidation impairs long-term spatial memory (Fig. 5e). Overall, our results show the transcriptional signature of spatial memory in the excitatory neurons of the RSC and further demonstrate the importance of activation of excitatory neuronal ensembles in the consolidation of long-term spatial memory.

## Discussion

In this study, we uncover the spatial transcriptomic signature of the RSC during spatial memory consolidation. Using state-of-the-art spatial transcriptomic approaches, we demonstrate the cell type and spatially restricted excitatory neuronal layer-specific signature of the learning-induced genes in the RSC. Lastly, using a chemogenetic approach, we show that inhibiting the activation of RSC excitatory neurons after learning impairs long-term spatial memory. Our study provides molecular insights into the transcriptional signature of the RSC in long-term spatial memory and reveals the signature of engram-related genes during an early time window of memory consolidation.

Spatial transcriptomics provides the ability to investigate brain regions at high spatial resolution. We first utilized our previously reported spatial transcriptomic dataset [27, 51] to study learning-induced gene expression changes within the RSC. We found that pathways related to transcriptional regulation (RNA Pol II specific DNA binding factor binding), protein folding (unfolded protein binding, protein folding chaperone, heat shock protein binding, misfolded protein binding) and MAP kinase (MAP kinase tyrosine/serine/threonine phosphatase activity, and MAP kinase phosphatase activity) were enriched in the RSC following learning. These findings were complemented with spatial transcriptomics at single-cell resolution using the Xenium platform. We have previously shown that learning induces the expression of several genes linked to the engram ensemble in the hippocampal CA1 pyramidal region [27]. Using the same data set, we found similar learning-induced changes in engram-specific IEG expression in the RSC. Similar findings were reported previously by *Baumgärtel* et al., where they showed gene expression from the hippocampus and RSC following contextual fear conditioning and object exploration tasks [42], however, these studies lacked single cell resolution. They found an overlapping gene induction pattern following learning in the RSC and the hippocampus. Consistent with this study, our unbiased spatial gene expression analysis using the Visium platform within the RSC following SOR training identified similar upregulation of IEGs such as *Arc*, *Fos*, *Egr2*, *Per1*, *Nr4a1*, *Egr1*, and *Fosb*. Comparable upregulation of activity-induced genes such as *Arc*, *Fos, and Egr1* in the RSC after learning in a spatial memory [66] or a contextual fear conditioning task has been reported in other studies [39, 40]. Elucidating the functional impact of each upregulated gene in the RSC after learning is challenging. A recent study described the role of one key learning-induced gene, *Per1* in long term memory consolidation. We found that *Per1* was upregulated in all the five major cell types after SOR learning in the RSC. Interestingly, knockdown of *Per1* in RSC or hippocampal neurons was shown to impair long-term memory in a spatial object location task [67–69]. Furthermore, studies have shown that lesions or inactivation of the hippocampus impair activity-dependent expression of IEGs *Arc*, *Fos*, and *Egr1* in the RSC [70, 71]. This may be indicative of the complex relationship between the hippocampus and the RSC, and the role of this circuit in learning-induced gene expression and spatial memory consolidation.

The central purpose of our current study was to examine the cell type-specific transcriptomic signature of memory-related gene expression in the RSC. To our knowledge, this is one of the first studies to uncover transcriptional profiling of the RSC at spatial and single-cell resolution during the early time window of memory consolidation. Our cell-type-specific spatial gene expression analysis using the Xenium platform revealed learning-induced expression of engram-specific IEGs in the different neuronal populations of the RSC. We found that classical engram-specific memory responsive, IEGs such as *Arc*, *Egr1*, and *Nr4a1* were induced in all five major cell types (neurons and glial populations), while genes such as *Dusp5*, *Fosb*, *Homer1*, and *Egr4* were induced only within neurons, suggesting the importance of studying learning-induced gene expression at single cell resolution. Our targeted spatial gene expression experiment using the Xenium platform allowed us to examine genes that were predominantly induced in spatially restricted classes of neurons. Following training in the SOR task, we expanded our spatial transcriptomics work to examine the selective induction of memory-responsive gene expression across anatomically distinct excitatory neuronal layers in the RSC. These layer specific excitatory neurons play a critical role on RSC connectivity [32] and behavior [72, 73]. Although most of the classical IEGs considered markers of engrams were not restricted to any particular cell types or layers, we still found some regional and cell type-specific induction patterns for a few learning-induced genes. *Cbln4* was exclusively upregulated following learning in excitatory neurons in layer 2/3. Cbln4 is a secreted synaptic protein associated with inhibitory synapse recruitment [74]. One study showed a similar expression pattern of *Cbln4* in L2/3 of the RSC and found it was critical for synaptic density, which would have important implications on neuronal activity in the RSC [75]. We also found memory-related gene *Gadd45a* was upregulated exclusively in layer L6 excitatory neurons after learning. Gadd45a has previously been shown to have a role in spatial memory consolidation [76]—loss of Gadd45a impairs memory, while overexpression of Gadd45a in the hippocampus improves memory [77]. Therefore, our work provides the framework for future research to investigate the mechanistic underpinnings of these layer specific changes in learning-induced gene expression.

Our spatial transcriptomic analysis at single-cell resolution identified the upregulation of several memory-related genes in all excitatory neuronal populations in the RSC. Using a deep learning computational tool, NEUROeSTIMator, we showed increased predicted activity in the RSC after learning. Interestingly, in a mutant tau model of ADRD (Tau-P301L) [53], which exhibits spatial memory impairment in the SOR task, we demonstrate tau pathology in the RSC. We also show decreased predicted activity of both excitatory and inhibitory neurons following learning. *Fos*, a marker of neuronal activity was downregulated in the excitatory neurons of the RSC after learning. These findings suggest that neuropathology in the RSC may affect learning-induced excitatory neuronal activation. Therefore, we employed a chemogenetic inactivation approach to investigate the role of excitatory neuron activation in the RSC after learning in adult mice. Our inhibitory DREADD approach successfully blocked the learning-induced activation of Fos, a commonly used marker of neuronal activity and engram ensembles. We found that inhibiting the excitatory neurons in the RSC after learning impairs spatial memory. This finding is consistent with previous research identifying key RSC neurons involved in spatial memory [73]. Thus, our work reveals that excitatory neuronal activation in the RSC during the early time window after learning is essential for long-term spatial memory consolidation. Previous inactivation studies have shown that the RSC and dorsal hippocampus are required for object location memory [49], with engrams being identified in these two brain structures [34, 41, 78]. However, the current theory of engrams in the field is that cortical and hippocampal engrams appear together, but those engrams must be matured to be engaged during retrieval [3, 41, 79, 80]. Interestingly, the RSC engrams generated after spatial learning are retained over prolonged periods, and the stability of those engrams during reactivation determines the strength of memory [41]. Therefore, understanding the engram-specific gene expression signature of the RSC during memory consolidation and its impact on behavior can provide us with valuable insights into neuronal dynamics that are crucial for the storage of long-term spatial memory.

We utilized two cutting-edge spatial transcriptomics approaches to delineate the learning-induced gene expression signature of the RSC. The 55 µm barcoded spot size of Visium provides a broad, unbiased transcriptomic analysis, while Xenium utilizes a targeted approach to investigate gene expression at single-cell resolution. The gene detection sensitivity of Visium is still lower than that of conventional transcriptomic approaches such as bulk RNA seq [81]. Thus, the low detection sensitivity of Visium could account for genes we identified to be induced by learning using Xenium but not Visium, such as *Bdnf* and *Gadd45a*. In conclusion, our study employed high-resolution spatial information to examine learning-induced genes in the RSC following SOR training. Even though our data is limited to a panel of genes, future work would investigate a larger pool of genes at single-cell resolution. Furthermore, the specific transcriptomic signature we identified across the laminar layers of the RSC suggests that each neuronal sub-type responds differently to activation related to memory consolidation. A recent study showed that IEG expression is reduced in layer 4 of the RSC in aged animals [82] which might provide the molecular basis of memory decline that is commonly seen with aging [83, 84]. Given the role of RSC inhibitory neurons in memory and AD [85], future work will also examine the different inhibitory neuronal populations in RSC involved in spatial memory consolidation. Taken together, our work demonstrates a cell-type specific signature of learning-induced genes in RSC, laying the groundwork for future research to study the cell-type and layer-specific gene expression after learning in diseases associated with memory loss such as neurodegenerative diseases and age associated memory decline.

## Methods and materials

### Data reporting

No statistical methods were used to predetermine sample size.

### Animals

Adult male C57BL/6 J mice were purchased from Jackson Laboratories for all experiments. Tau-P301L (rTg4510) male mice were obtained from Jackson Laboratories. This model harbored two transgenes: tTA driven by CaMKIIα promoter, and human tau P301L driven by TetO promoter. Control mice used for this study expressed only tTA driven by the CaMKIIα promoter. Mice had open access to food and water, and lights were maintained on a 12-h light/dark cycle. All behavioral experiments were conducted in 3- to 4-month-old C57BL/6 J male mice and in 4.5- to 5-month-old rTg4510 male mice. Animals were randomly assigned to conditions when applicable. No software for randomization was used. All experiments were conducted according to U.S. National Institutes of Health (NIH) guidelines for animal care and use and were approved by the Institutional Animal Care and Use Committee of the University of Iowa, Iowa.

### Xenium sample preparation and data acquisition

Brains were extracted from mice one hour after learning and or from homecage, then placed in −40 °C isopentane for five minutes to freeze the tissue, then stored at −80 °C. Mouse brains were embedded in OCT (Fisher Healthcare) and sectioned using a cryostat to 10 µm thick before being mounted on a Xenium slide (10x Genomics). Tissue was cut to mount only the RSC and hippocampus, which allowed for the placement of eight half-brain tissue samples per slide for further processing and analysis. All reagents used in Xenium sample preparation were purchased from 10x Genomics. Prior to probe hybridization, tissue was fixed in 4% paraformaldehyde for 30 min and then permeabilized using 1% SDS and chilled 70% methanol for 60 min. Slides were then placed into their cassettes and the pre-designed DNA probes were hybridized with the tissue overnight at 50 °C. 297 DNA probes were pre-designed by 10x Genomics- 247 included from their mouse brain gene expression panel, and 50 custom probes. After probe hybridization, samples were washed for 30 min followed by incubation with the ligation mix for two hours at 37 °C. Immediately after, samples were washed then incubated for additional two hours at 30 °C with the amplification master mix. Following another wash, samples were incubated overnight at 4 °C in buffer before autofluorescence quenching and nuclei staining the following days. Slides were processed in the Xenium Analyzer. Briefly, fluorescent probes were hybridized to their targets, imaged, and then washed off to allow for subsequent probe cycles. Imaging detected puncta, which were decoded into gene ID’s and assigned a quality score. All data was acquired and further processed using R.

### Xenium analysis

#### Data preprocessing

The Xenium Ranger output files were imported into R (4.3.1) and a Seurat spatial transcriptomics object was created using the LoadXenium function (Seurat package v5.1.0). The data was filtered to remove cells with zero mRNA counts (using nCount_Xenium > 0). Subsequently, a region of interest (ROI) around the RSC was lassoed from the coronal section image (using Xenium explorer v3). This selected the RSC cells to be used for creating a Seurat object. The same process was followed for all biological replicates. The data from homecage control and SOR was normalized (using SCTransform) and integrated into a single Seurat object (using Seurat based FindIntegrationAnchors function). UMAP embeddings for the integrated object was computed using 30 principal components (RunPCA(npcs = 30), and RunUMAP(dims=1:30)). A k-nearest neighbor graph was constructed between every cell based on Euclidean distance in PCA space using FindNeighbors after which the unsupervised cell clustering was performed using the original Louvain algorithm (FindCluster (resolution=0.1)). We identified 16 clusters initially and further subclustered the inhibitory neuron clusters to increase the resolution. Cluster-specific markers were identified (using FindAllMarkers (logfc.threshold = 0.20)) and clusters were manually annotated from curated gene sets in the Xenium Panel. Two rounds of celltype annotations were performed. In the first round, major cell types identified were: excitatory neurons, inhibitory neurons, astrocytes, oligodendrocytes, microglia, endothelial cells, oligodendrocyte progenitor cells, and Vacuolar leptomeningeal cell (VLMC) were identified. In the second round, excitatory neurons were further classified into seven subtypes according to the cortical layers, inhibitory neurons were further clustered into five subtypes based on the markers *Pvalb, Sst, Lamp5, Sncg, Vip*. Cells were visualized using the Seurat based ImageDimPlot function. For the experiment comparing Tau P301L-learning and control-learning, brain sections from one Tau-P301L mouse was excluded due to poor tissue quality. We used the same data normalization, integration, and clustering method as mentioned before. Major cell types were identified based on marker gene expression.

#### Differential gene expression analysis

The differential gene expression analyses were performed to compare gene expression between (i) learning and homecage control conditions and (ii) Tau-P301L learning and control learning conditions across different celltypes (using FindMarkers function), with the following parameters: min.pct=0.2, logfc.threshold=0, test.use = “wilcox”, assay = “SCT”. Genes with an |log2 fold change| > 0.2 and adjusted p value < 0.05 were considered significant. To compare differentially expressed genes (DEGs) between Fos⁺ and Fos⁻ neurons (both excitatory and inhibitory) within the learning group, we first identified Fos⁺ cells as those with *Fos* expression > 0. Cells with no detectable *Fos* expression were annotated as Fos⁻. Upon aggregating all biological replicates from the learning condition, we found that approximately 31.45% of cells were Fos⁺, while 68.55% were Fos⁻. Next, we examined *Fos* expression within major neuronal subtypes. Among excitatory neurons, 51.28% were Fos⁺, whereas 39.28% of inhibitory neurons showed *Fos* positivity. Differential gene expression analysis between Fos⁺ and Fos⁻ neurons was then conducted separately for excitatory and inhibitory populations using the same parameters as previously described.

### Molecular function enrichment analysis

The significant DEGs (p_val_adj < 0.05 & abs(avg_log2FC) > 0.2), including both upregulated and downregulated genes were further subjected to enrichment analysis executed using clusterProfiler v4.10. The Gene Ontology (Molecular Function) enrichment was performed using the enrichGO function implemented in clusterProfiler package with the following parameters: pvalueCutoff = 0.01, qvalueCutoff = 0.05, pAdjustMethod = “BH”, ont = “MF”. Further data visualizations were done using the clusterProfiler package.

#### NEUROeSTIMator

We used NEUROeSTIMator [51], a deep learning model trained to infer neuronal activation from gene expression data, to quantify neuronal activity across cell types and classes for each condition (e.g. wild-type learning, wild-type homecage, Tau P301L-learning, and Control-learning). Predicted activity was estimated at the single-cell level using the transcriptomic profile of the 297 genes. To assess differences in activity levels between conditions, we fit a logistic regression (LR) model for each cell type and extracted the ß coefficient and p-value for predicting the experimental condition. The reported estimates correspond to prediction of the (i) learning group in the wild-type experiment, and (ii) the Tau P301L-learning condition in the experiment comparing Tau P301L-learning and control-learning groups.

### Statistics

All data from behavioral studies, qPCR and immunohistochemistry were analyzed and visualized using GraphPad Prism. Sample size was estimated based on previous studies. No statistical software was used to predetermine sample size. Animals were assigned to the experimental groups randomly. The data normality and variance were tested using Shapiro-Wilk normality test. Normally distributed data were analyzed using unpaired two-tailed t-tests and two-way ANOVAs with repeated measures as within-subject variable. Sidak’s multiple comparison tests were used for post hoc analyses where needed. Differences were considered statistically significant when p  <  0.05.

Methods are fully detailed in the Supplemental Information.

## Supplementary information

Supplemental information

Supplementary Table 1

Supplementary Table 2

Supplementary Table 3

Supplementary Table 4

Supplementary Table 5

Supplementary Table 6

Supplementary Table 7

## Data Availability

The Xenium data generated in this study have been deposited in the Zenodo repository 10.5281/zenodo.16697913.

## Code availability

The code used for Xenium and Visium data analysis can be accessed through GitHub https://github.com/ChatterjeeEpigenetics/RSC_learning_2025.
