# Supplementary material for: Single-cell resolution spatial transcriptomic signature of the retrosplenial cortex during memory consolidation
Source: Mol Psychiatry. 2025 Nov 4;31(4):1845–56. doi: 10.1038/s41380-025-03331-3 (PMC12645481; doi:10.1038/s41380-025-03331-3)
Supplement: Supplementary file 1 — Supplemental information [file 41380_2025_3331_MOESM1_ESM.docx]

**Single-cell resolution spatial transcriptomic signature of the retrosplenial cortex during memory consolidation**

Savannah R. Bliese^1,2,3#^, Budhaditya Basu^1,2#^, Stacy E. Beyer^1,2^, Muhammad Elsadany^4^, Jacob J Michaelson^4^ and Snehajyoti Chatterjee^1,2*^

^1^ Department of Neuroscience and Pharmacology, Carver College of Medicine, University of Iowa, Iowa City, IA, USA 52242

^2^ Iowa Neuroscience Institute, University of Iowa, Iowa City, IA, USA 52242

^3^ Interdisciplinary Graduate Program in Neuroscience, University of Iowa, Iowa City, IA, USA 52242

^4^ Department of Psychiatry, University of Iowa Hospitals and Clinics, Iowa City, IA, USA 52242

^#^Authors contributed equally

*Corresponding author:

snehajyoti-chatterjee@uiowa.edu

**Keywords:** Retrosplenial cortex, spatial memory, memory consolidation, gene expression, spatial transcriptomics.

**Running title:** Gene Expression in RSC and Spatial Memory

This file includes Supplementary methods, Supplementary Figures, Supplementary Figure Legend 1-10 and Supplementary Table legends 1-7.

**Supplementary materials and methods**

***Drugs:*** Clozapine-n-oxide (abcam, ab141704) was dissolved in 0.9% sterile saline. For behavioral experiments, mice were injected intraperitoneally with a dose of 2.5 mg/kg immediately following training. 0.9% sterile saline was used as vehicle control.

***Adeno-Associated Viral (AAV) Constructs and Stereotaxic Surgeries:*** pAAV-CaMKIIa-hM4D(Gi)-mCherry was a gift from Bryan Roth (Addgene viral prep # 50477-AAV2). Mice were anesthetized with 5% isoflurane (Piramal Critical Care) for induction and then maintained at 2.0-2.5% during the surgical procedure. Betadine (Andwin Scientific) and ethanol pads were used to sterilize the scalp; then an incision was made down the midline to expose the skull. Holes were drilled into the skull above the RSC. The following coordinates (from Bregma) were used for viral injection into the RSC: 1.8 mm posterior, 0.45 mm lateral, and 0.75 mm ventral. A 33-gauge needle (WPI) attached to a 10 µl syringe was lowered into the RSC at a rate of 0.1 mm/10 sec. The needle rested at 0.65 mm below bregma for one minute then advanced down to 0.75 mm. Bilateral injections of the AAV constructs, 1 µl per side, were carried out at a rate of 100 nl/min, which was controlled by a micro syringe pump (UMP3; WPI). After the total volume was injected, the needle remained in place for an additional 2 minutes. The needle was raised slightly, then allowed to rest for an additional one minute before it was raised at a rate of 0.1 mm/15 sec. Bone wax (Surgical Specialties) was used to fill in the drill holes, and sutures were used to close the incision. Meloxicam at a dose of 5mg/kg was administered as an analgesic and was given at the beginning of surgery and approximately 24 hours post-procedure.

***Spatial object recognition (SOR) task*:** Mice were individually housed for seven days before the start of behavioral testing. The SOR task was carried out as previously described ^1^. Animals were handled for two minutes a day for five days total. Behavioral experiments were carried out from ZT0 to ZT3. Animals were brought into the room at least 15 minutes prior to experiment start. First, animals were allowed to explore the arena for 6 minutes for a habituation trial. Three unique objects were then placed into the arena and three training trials of six minutes each were carried out with an intertrial interval of 5 minutes. 24 hours later, a single object was moved to a new location within the arena, and animals were tested for long-term spatial memory with a six-minute trial. Ethovision (Noldus) software was used to ensure arena settings remained the same between animals. Trials were manually scored by an investigator blind to the experimental group, and the amount of time the mouse investigated each object was recorded. To determine the percent preference for the displaced object, we took the total time spent exploring the moved object, divided by the total time exploring all objects, and multiplied that number by 100. Animal that showed an initial preference towards an object during training was removed from all analyses. Outliers were excluded if calculated as greater than two standard deviations from the mean. For all spatial transcriptomics experiments, animals were still single-housed for seven days and handled for five days ^1^. On the day of training, animals who were assigned to homecage condition remained in their cage, while animals assigned to the SOR learning condition carried out the habituation and training trials as described above. Whole brains or RSC tissue for these experiments were taken one hour after training, flash frozen, and then stored at -80C.

***Immunohistochemistry*:** Tissue for immunofluorescence staining and analysis was taken one hour after either the training or test session. Transcardial perfusions were performed using 4% paraformaldehyde (PFA, Sigma Aldrich) to fix brain tissue for preservation. Brians were removed and placed into 4% PFA for 24 hours. They were then placed in 10%, 20% and 30% sucrose solutions for 24 hours each, or until the tissue had sunk. Tissue was sliced into 14-20 µm thick sections using a cryostat (Epredia). For tissue stained with FOS, slices were blocked with 5% normal goat serum (Jackson Immuno, cat. # 005-000-121) for one hour before being incubated 24 hours at 4°C with a Fos antibody (SYSY Antibodies, cat. # 226 308, 1:5000) diluted in 0.4% PBS with Triton-X. The following day slices were washed in 1x PBS three times then incubated in an AlexaFluor 647 secondary antibody (Jackson Immuno, cat. # 106-605-003, 1:500) for two hours at room temperature (RT). RSC tissue sections from rTg4510 mice were collected and treated in similar conditions prior to the staining protocol. Slices were blocked with 10% normal donkey serum (Lampire, cat. # 50-413-115) for one hour before incubation with a phosphorylated human tau antibody (AH36, StressMarq Biosciences, cat. #SMC-601D, 1:500) diluted in 0.2% TBS with Triton-X overnight at 4°C. The following day slices were washed in 1x TBS three times then incubated in AlexaFluor 594 (Jackson Immuno, cat. # 711-585-152, 1:500) for two hours at RT.

***Image Acquisition and Analysis:*** Stained tissue was mounted onto Superfrost Plus microscope slides (Fisherbrand) and coverslipped using Prolong Diamond Antifade Mounting Medium with DAPI (Invitrogen). Images of the RSC were taken with a 20x air objective using the VS200 Slide Scanner (Olympus). All images taken for phosphorylated tau quantification and for FOS^+^ cell count used the same exposure time, laser, and gain settings within their respective conditions. Images taken for phosphorylated tau quantification were imported into ImageJ for mean fluorescence intensity (MFI) analysis. The region of interest, the RSC, was determined and outlined using the polygon tool. The channels were split to isolate the phosphorylated tau fluorescent channel, which was then adjusted to an 8-bit greyscale image. The subtract background tool was used to reduce any signal from autofluorescence. From here, the mean fluorescence intensity was determined using the measure tool. All data was normalized to the average phosphorylated tau expression of the Tau- control mice. Images taken for FOS^+^ cell counts were imported into QuPath for cell count analysis. Briefly, region of interest was determined for the RSC and total cell count was found using the DAPI counterstain. Then the positive cell detection for mCherry and FOS were determined using predetermined thresholds. For FOS, the threshold was determined to be 200 percent of the mean fluorescence value of the background. Counts were then imported into Excel to find the percent of cells expressing FOS for both mCherry^+^ cells and total cells, and the results were analyzed using Prism software.

**Visium spatial transcriptomics data analysis:** Count matrices from the previous published data (GSE223066 and GSE201610)^1, 2^ were loaded into R (version 4.3.1) and a Seurat object was created using Load10X_Spatial function (Seurat package, version 5.1.0). Using Loupe Browser v8, we selected the Visium spots covering the RSC region and barcodes were exported as CSV files. The spatial barcodes were used to subset the RSC area from the whole coronal section. SCTransform normalization was performed on each replicate separately. Replicates from homecage controls and SOR samples were then integrated using a Seurat integration pipeline. Briefly, the integration anchors were found (FindIntegrationAnchors) from the list of 14 Seurat objects of homecage control and SOR samples. These anchors were used to integrate the 14 datasets together (IntegrateData). Principal component analysis (PCA) was performed using the RunPCA function (npcs=30). Based on 30 PCs, UMAP reduction was derived (using RunUMAP function). A k-nearest-neighbours graph was constructed based on Euclidean distance in PCA space and unsupervised Visium spots clustering was performed using a Louvain algorithm implemented in the Seurat package (FindNeighbors and FindClusters functions).

*Pseudobulk differential gene expression analysis*: Instead of cluster based differential expression analysis, all Visium spots across the seven biological replicates from learning group were pooled as pseudo bulk learning group and spots from homecage (HC) controls across the seven biological replicates were pooled as pseudo bulk homecage group. The pseudo bulk differential gene expression between learning and homecage was performed using the FindMarkers function with the following parameters: min.pct=0.2, test.use = "wilcox". Each Visium spot was used as a replicate in this pseudo bulk analysis. The volcano plot and Sankey plot were generated using a custom ggplot2 script. Genes with a |log2 fold change| > 0.2 and adjusted p value < 0.05 were considered significant.

***Total RNA extraction, cDNA preparation, and qPCR analysis*:** RSC tissue was extracted from animals one hour after learning on a spatial object recognition task and placed in RNAlater (Invitrogen) at -80° C. For RNA extraction, tissue was homogenized in Qiazol (Qiagen) with stainless steel beads (Qiagen), then added to chloroform and centrifuged at 4°C at 12,000g for 15 minutes. The aqueous solution was collected and added to EtOH, and subsequently washed using buffers and nuclease free water from the Qiagen RNAeasy Kit in a column tube (Qiagen). Samples were treated with DNase for 25 minutes at room temperature. Next, 100% EtOH, sodium acetate and glycogen were added to samples and allowed to rest for one hour at -20°C before being centrifuged at top speed for 20 minutes at RT. Supernatant was removed and 80% EtOH was added to the sample and spun again for five minutes before aspirating the remaining EtOH. Samples were allowed to dry before being resuspended in nuclease free water. RNA concentration was estimated using a Nanodrop (ThermoFisher), and estimates were used to produce a solution containing 1 µg of RNA. cDNA was prepared using the SuperScript™ IV First-Strand Synthesis System (Invitrogen) and diluted to 2 ng/µl. 2.25 µl of each sample was added to a 384 well plate along with 2.5 µl of Fast SYBR™ Green Master Mix (ThermoFisher) and 0.25 µl of a 5 µM primer mix (IDT). Real-time PCR was carried out using a QuantStudio 7 Flex Real-Time PCR System (Applied Biosystems, Life Technologies). Samples were run in triplicates and all data was normalized to housekeeping genes. The 2^(-ΔΔCt)^ method was used for gene expression analysis. The following sequences were used to create custom primers for use with qPCR: *Egr1* (FW: 5’ TGAACAACGAGAAGGTGCTG 3’; Rev: 5’ AGCGGCCAGTATAGGTGATG 3’), *Nr4a1* (FW: 5’ AAAATCCCTGGCTTCATTGAG 3’; Rev: 5’ TTTAGATCGGTATGCCAGGCG 3’), *Dusp5* (FW: 5’ GACAGCCACACTGCTGACAT 3’; Rev: 5’ AGGACCTTGCCTCCTTCTTC 3’ ), *Tubulin* (FW: 5’ ATGCGCGAGTGCATTTCAG 3’; Rev: 5’ CACCAATGGTCTTATCGCTGG 3’), *Pgk1* (FW: 5’ CGAGCCTCACTGTCCAAACT 3’; Rev: 5’ TCTGTGGCAGATTCACACCC 3’), *Actb* (FW: 5’ TCAACACCCCAGCCATGTAC 3’; Rev: 5’ CGGAGTCCATCACAATGCCT 3’).

**References**

1. Vanrobaeys Y, Mukherjee U, Langmack L, Beyer SE, Bahl E, Lin LC *et al.* Mapping the spatial transcriptomic signature of the hippocampus during memory consolidation. *Nat Commun* 2023; **14**(1)**:** 6100.

2. Bahl E, Chatterjee S, Mukherjee U, Elsadany M, Vanrobaeys Y, Lin LC *et al.* Using deep learning to quantify neuronal activation from single-cell and spatial transcriptomic data. *Nat Commun* 2024; **15**(1)**:** 779.

**Supplementary Figures**

**
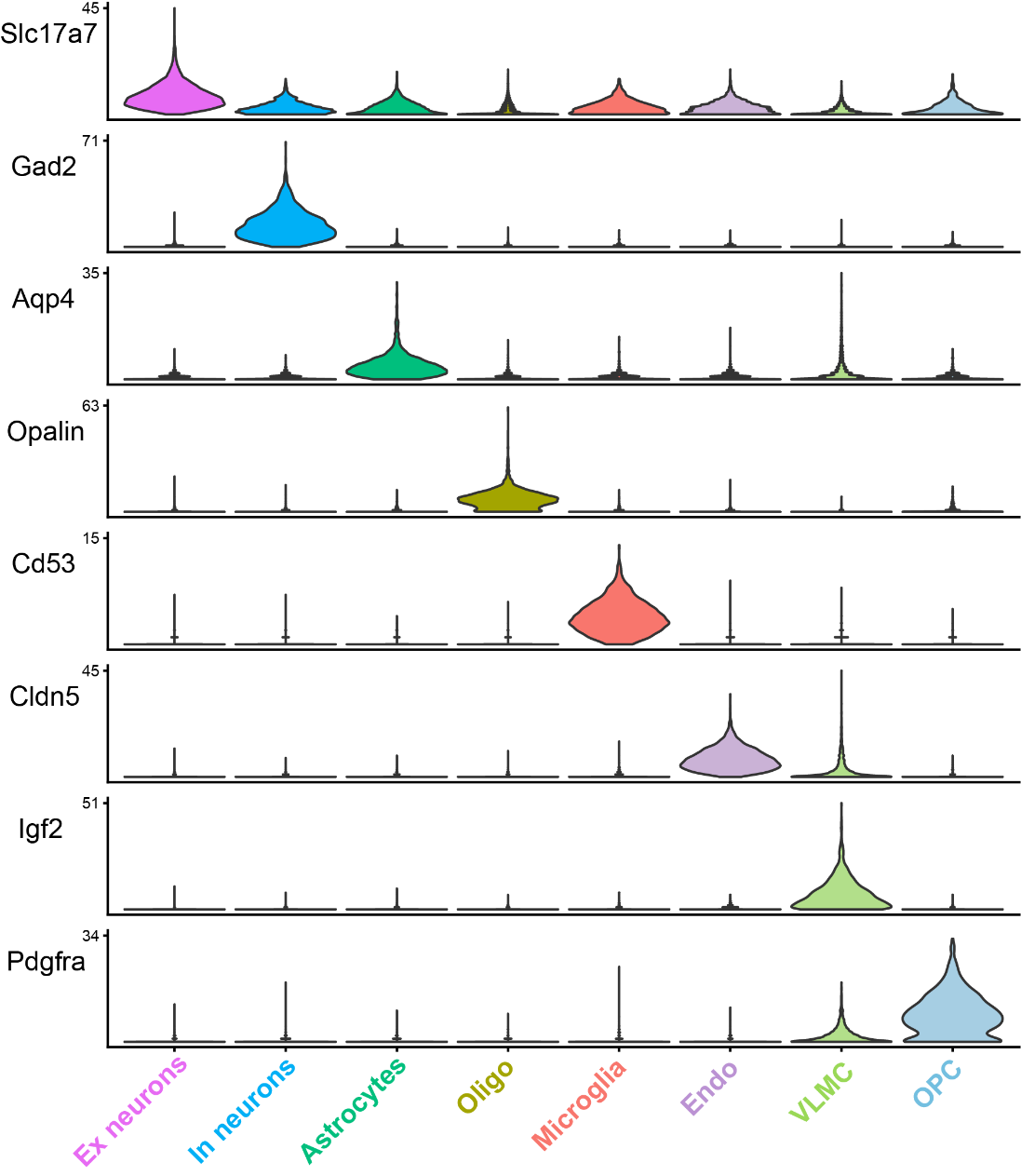
**

**Supplementary Figure 1.** **Expression of marker genes for major cell types within RSC**

Violin plot depicting the expression of marker genes identified in the learning and homecage control groups using Xenium spatial transcriptomics. Ex neurons, excitatory neurons; In neurons, Inhibitory neurons; Oligo, oligodendrocytes; Endo, endothelial cells; VLMC, vascular leptomeningeal cells; OPC, oligodendrocyte progenitor cells.

**
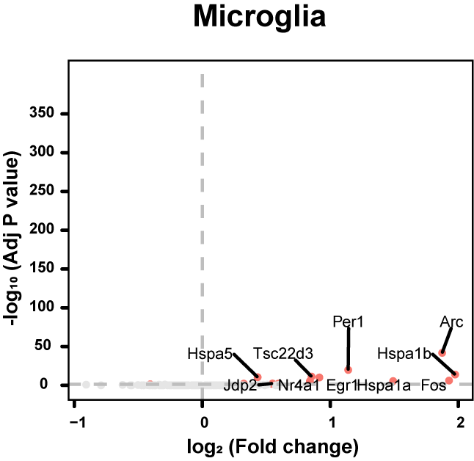
**

**Supplementary Figure 2. Learning-induced differential gene expression in microglia.** Volcano plot showing significant differentially expressed genes (FDR <0.05, log2 foldchange threshold = ±0.2) in microglia of adult mice within RSC region.


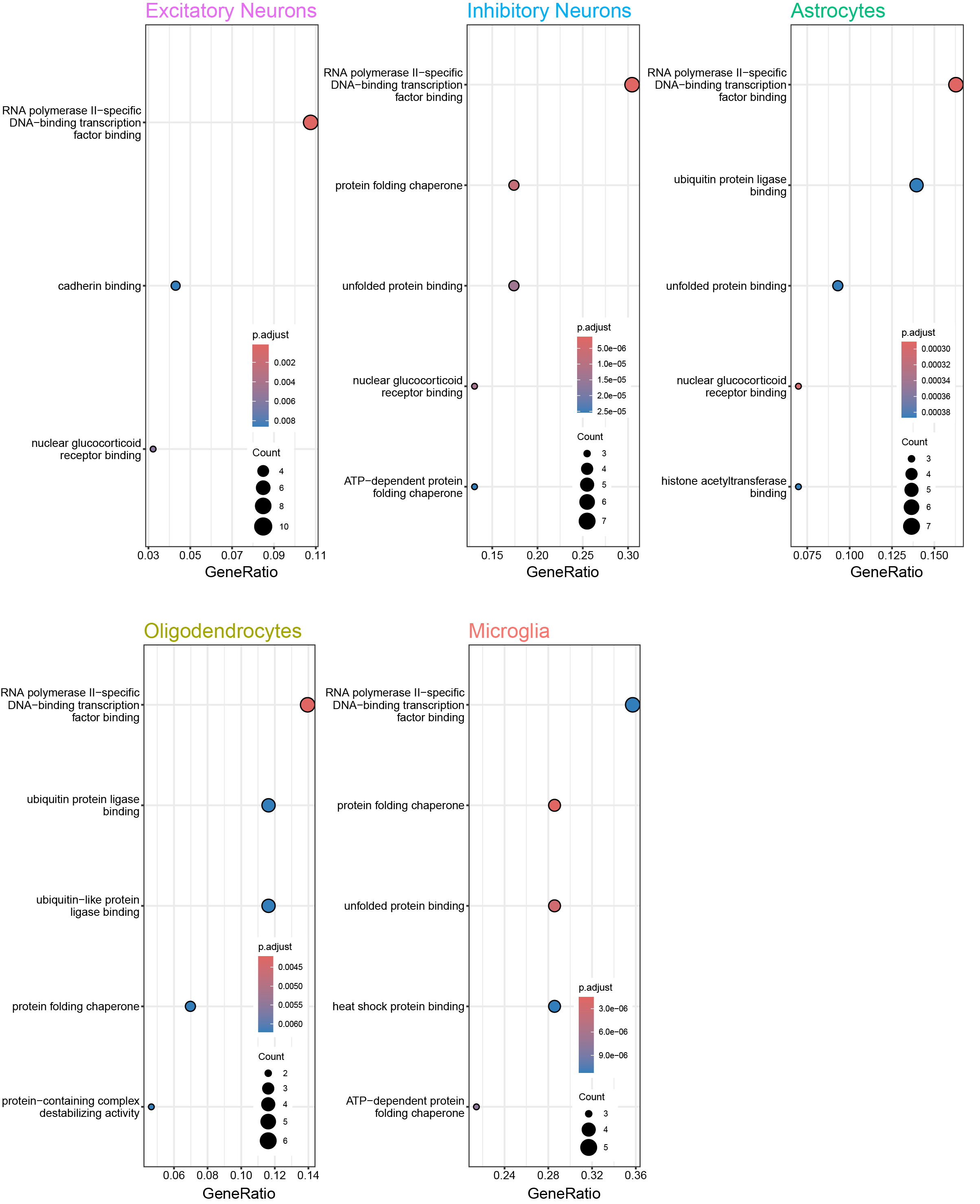


**Supplementary Figure 3.** **Gene ontology (GO: molecular function) enrichment analysis across five major cell types.** Top five enriched functions are depicted in the dot plot. GeneRatio implies the proportion of genes found to be differentially expressed within a specific GO term.

**
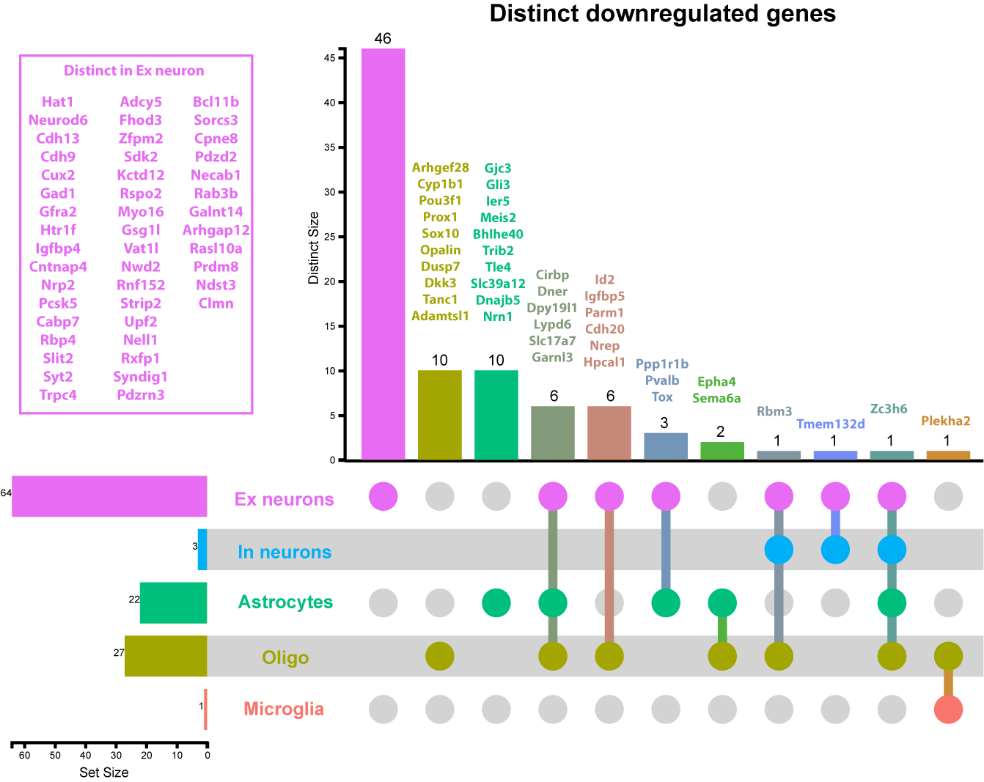
**

**Supplementary Figure 4. Distinct and overlapping downregulated genes after learning** **across five major cell types.** UpSet plot depicting all the significantly downregulated genes in the RSC across the five major cell types (excitatory neurons, inhibitory neurons, astrocytes, oligodendrocytes, and microglia) using the Xenium approach.


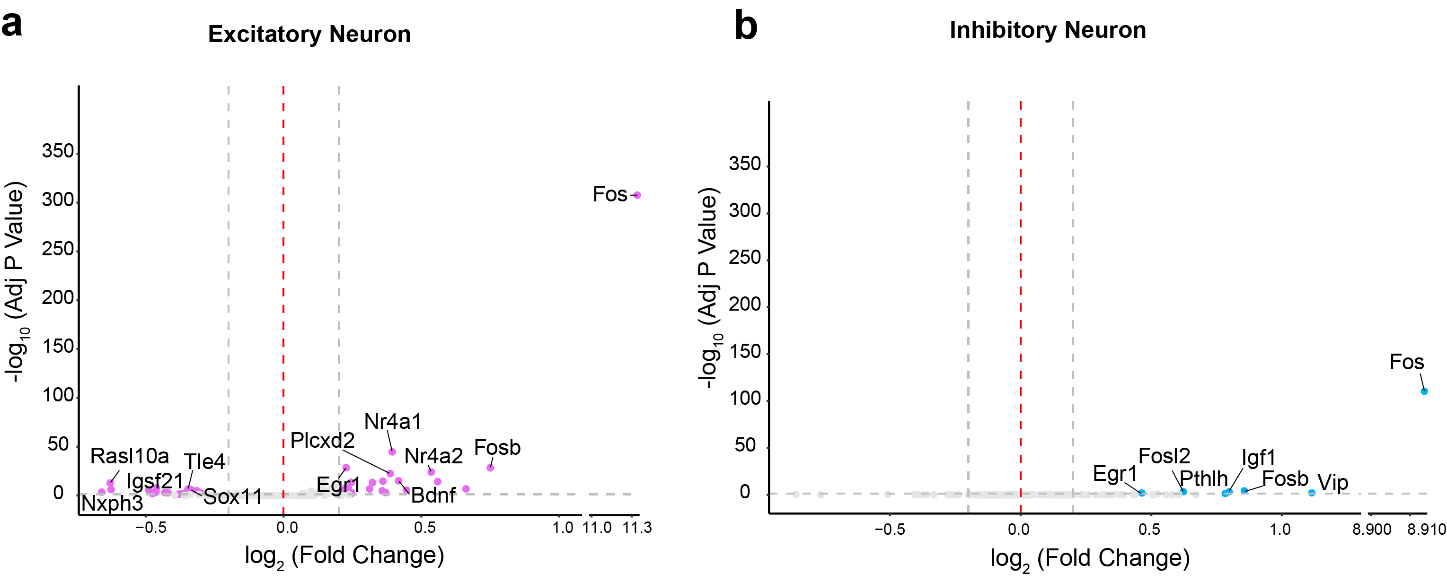


**Supplementary Figure 5.** **Gene expression signature changes in Fos+ neurons compared to Fos- neurons in the RSC of adult mice.** Volcano plot showing significant differentially expressed genes (FDR <0.05, log2 foldchange threshold = ±0.2) in Fos+ neurons compared to Fos- neurons in SOR trained samples **a.** excitatory neurons, **b.** inhibitory neurons.


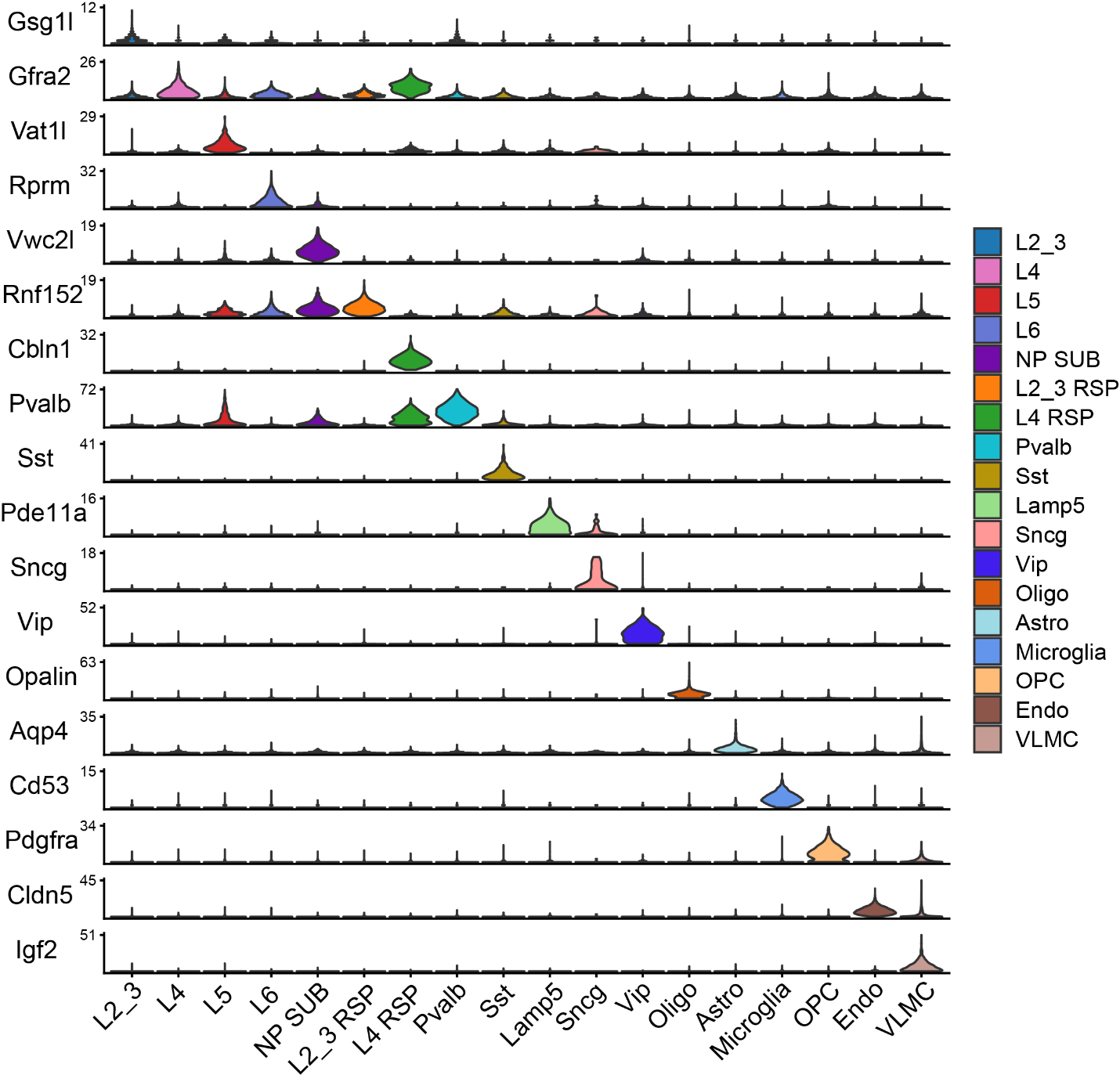


**Supplementary Figure 6.** **Expression of marker genes for layer-specific neuronal subtypes within RSC.** Violin plot depicting the expression of marker genes for each cell type including layer-specific neuronal subtypes identified within RSC of learning and homecage control groups using Xenium spatial transcriptomics

**
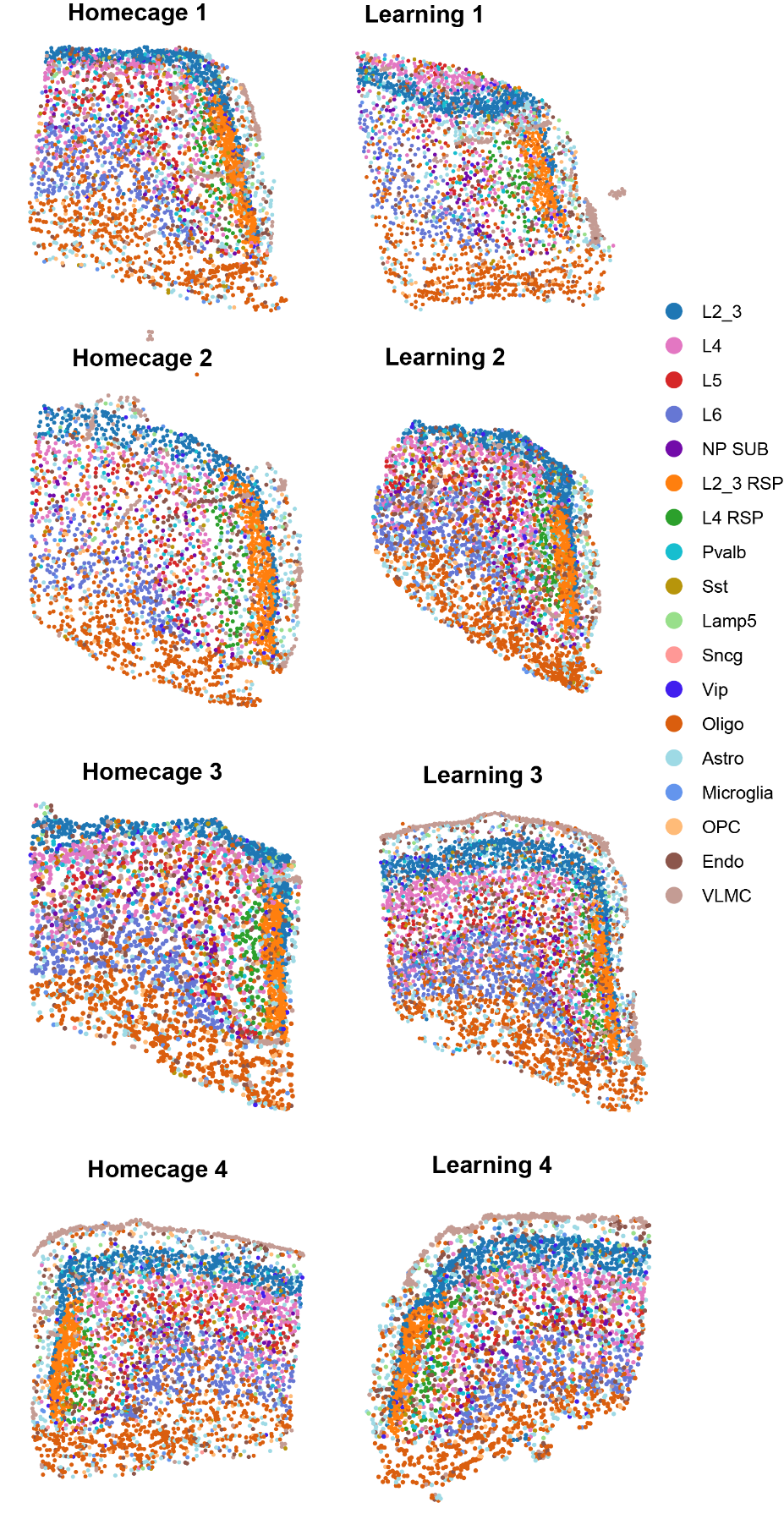
**

**Supplementary Figure 7.** Spatial positions of cell subtype within the RSC across the biological replicates of learning and homecage groups.


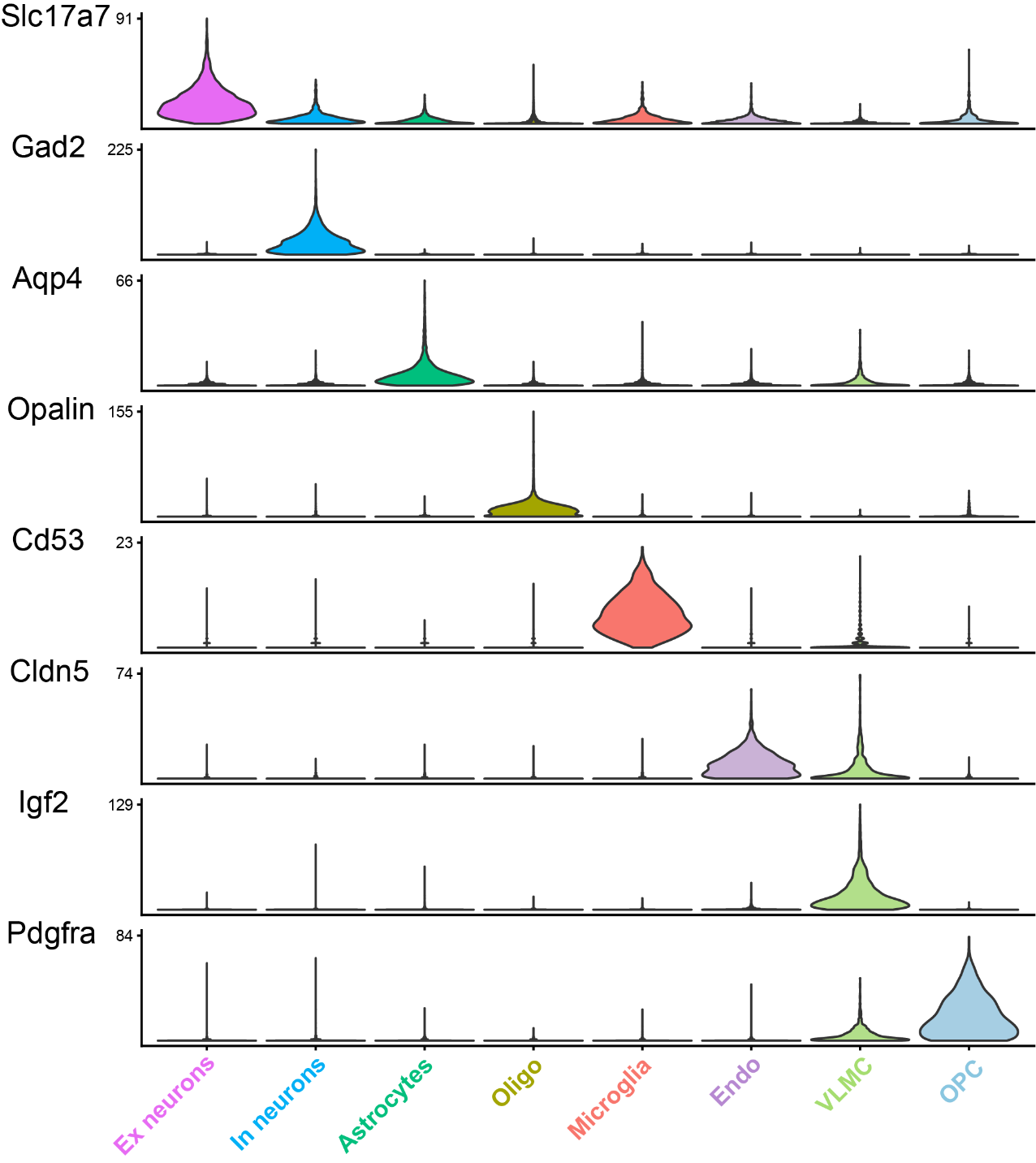


**Supplementary Figure 8.** **Expression of marker genes for major cell types within RSC of Tau-P301L learning and control learning** **groups**. Violin plot depicting the expression of marker genes for each cell type (Tau-P301L learning and control learning).

**
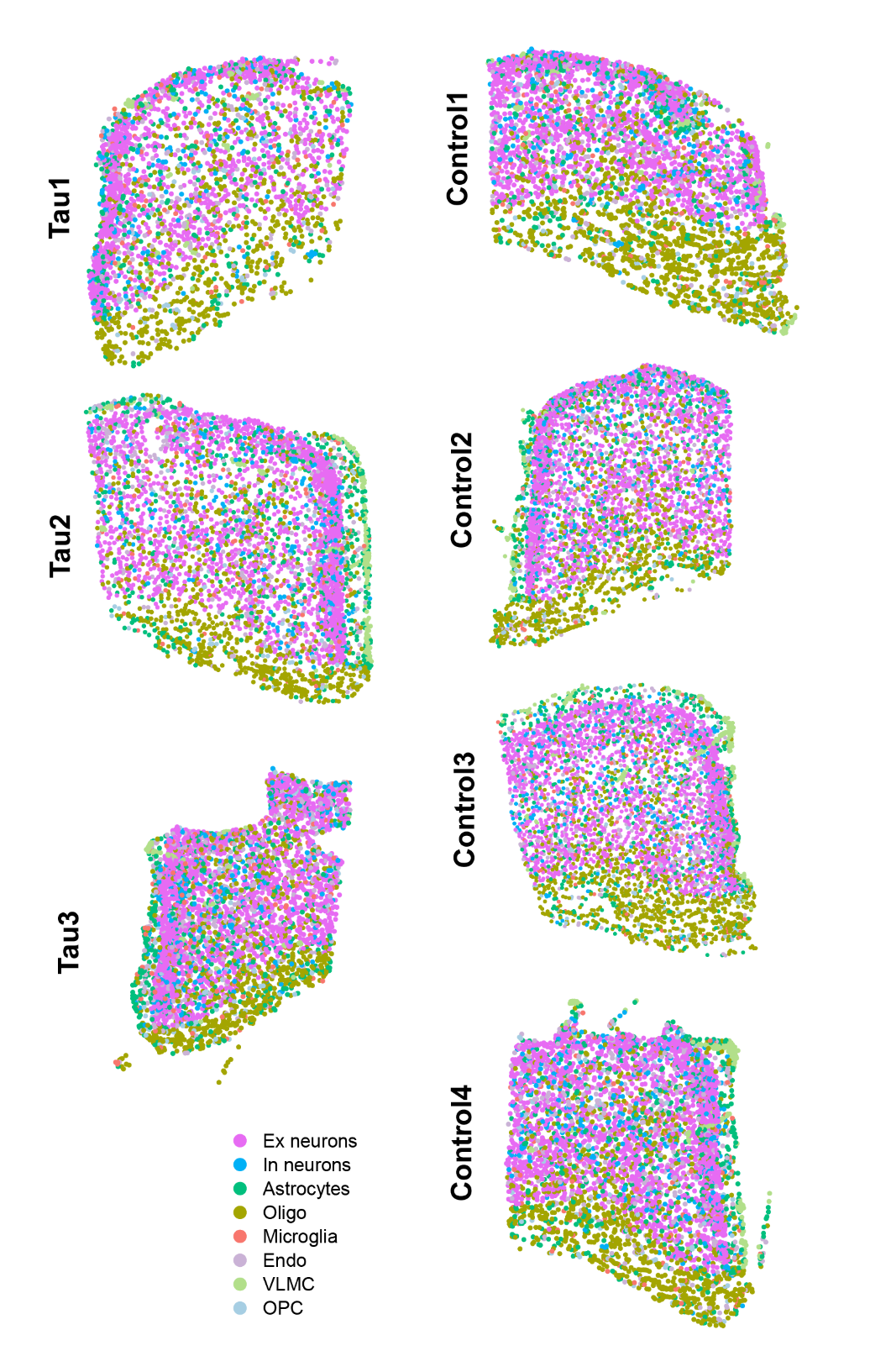
**

**Supplementary Figure 9.** Spatial positions of major cell types identified within the RSC across the biological replicates of tau P301L learning and control learning groups.


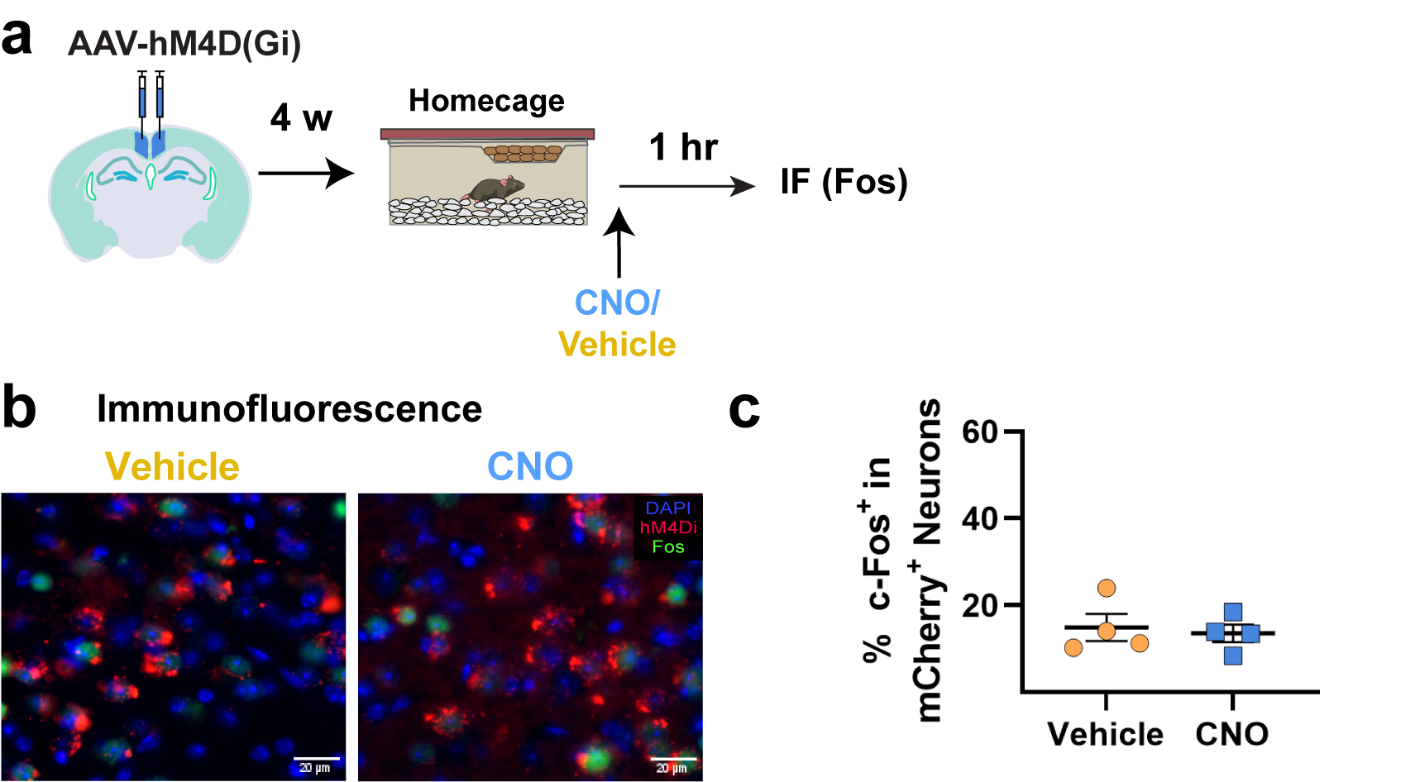


**Supplementary Figure 10. Chemogenetic inhibition of excitatory neurons in homecage mice. a.** Schematics of the experiment. **b.** Representative immunofluorescence images demonstrating Fos protein expression within DREADD positive neurons for the saline and CNO treated groups. **c.** Dot plot showing the percent of DREADD positive neurons which are also positive for Fos protein expression 1 hour after CNO administration. The data is shown as mean ± SEM.

**Supplementary Table Legends**

**Supplementary Table 1:** List of DEGs from RSC after learning in adult mice using Visium spatial transcriptomics.

**Supplementary Table 2:** GO (Molecular Function) enrichment analysis on learning-induced DEGs identified by Visium spatial transcriptomics.

**Supplementary Table 3:** A 297-probe panel used for Xenium spatial transcriptomics which includes marker genes for cell type identification and 50 custom genes.

**Supplementary Table 4:** List of DEGs from the major cell types identified within RSC after learning using Xenium spatial transcriptomics. Ex neurons; In neurons, inhibitory neurons; Oligo, oligodendrocytes; Endo, endothelial cells; VLMC, vascular leptomeningeal cells; OPC, oligodendrocyte progenitor cells

**Supplementary Table 5:** GO (Molecular Function) enrichment analysis on learning-induced DEGs in major cell types within RSC as identified by Xenium spatial transcriptomics.

**Supplementary Table 6:** List of DEGs comparing Fos^+^ and Fos^-^ neurons (excitatory and inhibitory) in the RSC following learning.

**Supplementary Table 7:** List of DEGs from the layer-specific excitatory neuronal and inhibitory neuronal subtypes in the RSC following learning as identified by Xenium spatial transcriptomics.
